# Relief-oriented use of marijuana by teens

**DOI:** 10.1186/1747-597X-4-7

**Published:** 2009-04-23

**Authors:** Joan L Bottorff, Joy L Johnson, Barbara M Moffat, Tamsin Mulvogue

**Affiliations:** 1 Centre for Healthy Living and Chronic Disease Prevention, University of British Columbia Okanagan, 3333 University Way, Kelowna, BC V1V 1V7, Canada; 2 NEXUS Research Unit, University of British Columbia, 302-6190 Agronomy Road, Vancouver, BC V6T 1Z3, Canada; 3 School of Nursing, University of British Columbia, 302-6190 Agronomy Road, Vancouver, BC V6T 1Z3, Canada

## Abstract

**Background:**

There are indications that marijuana is increasingly used to alleviate symptoms and for the treatment of a variety of medical conditions both physical and psychological. The purpose of this study was to describe the health concerns and problems that prompt some adolescents to use marijuana for therapeutic reasons, and their beliefs about the risks and benefits of the therapeutic use of marijuana.

**Methods:**

As part of a larger ethnographic study of 63 adolescents who were regular marijuana users, we analyzed interviews conducted with 20 youth who self-identified as using marijuana to relieve or manage health problems.

**Results:**

Thematic analysis revealed that these teens differentiated themselves from recreational users and positioned their use of marijuana for relief by emphasizing their inability to find other ways to deal with their health problems, the sophisticated ways in which they titrated their intake, and the benefits that they experienced. These teens used marijuana to gain relief from difficult feelings (including depression, anxiety and stress), sleep difficulties, problems with concentration and physical pain. Most were not overly concerned about the risks associated with using marijuana, maintaining that their use of marijuana was not 'in excess' and that their use fit into the realm of 'normal.'

**Conclusion:**

Marijuana is perceived by some teens to be the only available alternative for teens experiencing difficult health problems when medical treatments have failed or when they lack access to appropriate health care.

## Background

There is lively public debate surrounding the use of medical marijuana. While some remain sceptical about the therapeutic value of marijuana, there is a growing body of research that emphasizes its salutary effects. The literature points to the use of marijuana among adults to alleviate a variety of symptoms including pain, nausea, muscle spasm, insomnia, anorexia and anxiety as well as the treatment of a variety of medical conditions that are both physical and psychological [1-5]. However, less is known about adolescents' use of marijuana for therapeutic purposes.

### Background Literature

For individuals who set out to "feel better" through the use of marijuana, use has also been referred to as "self-medication," a hypothesis which posits that people do not misuse substances solely for the experience of being "high;" rather, they do so as a means of gaining relief from psychological and emotional pain [6]. In contrast to the adult literature on marijuana use where therapeutic use is linked to treatment of specific symptoms and illnesses, in the adolescent literature there is less clarity about how to define non-recreational uses of marijuana.

A motivationally-driven approach is one way that researchers have attempted to understand marijuana use among adolescents [7]. It is proposed that different reasons for using marijuana may shape patterns and contexts of use, which in turn may be associated with different problems related to use. Social motives for marijuana use, for example, have been associated with patterns of recreational use (e.g., sensation seeking). Coping motives have been used to classify adolescents using marijuana for non-recreation purposes. Differences have been observed among youth using marijuana for social and coping reasons that support the motives framework. In contrast to youth aged 16–24 years using marijuana for social reasons, users of the same age reporting coping motives have been observed to have lower mental health, higher psychopathology, more psychosocial distress and more stressful life events than non-cannabis-using youth [8].

Although there is a large body of literature related to recreational use of marijuana among adolescents [9,10] less is known about other motivations for the use of marijuana in this population. Several hypotheses have emerged related to non-recreational uses of marijuana among adolescents. The "self-medication hypothesis" [11-14] is most closely associated with the therapeutic use of marijuana. Instrumental use is another term applied to taking drugs for specific pharmacological effects of the substance rather than for pleasure or recreational purposes. For example, Glassner [15] examined the notion of instrumental drug use in a qualitative study of young drug users, and found that marijuana was used for its calming effects, to relieve tension, and to gain self-confidence. Further, support for a typology of drug related beliefs that included relief-oriented beliefs [16] was demonstrated in a study of cannabis use in a sample of 285 French high school students [17]. In this study, four 'positive' relief-oriented beliefs were identified related to how the substance creates relaxation and calms anxiety, reduces suffering, relieves boredom, and makes one feel better. The presence of relief-oriented beliefs was the only predictor of cannabis dependence.

Associations between marijuana and psychological problems have also lead researchers to consider other possible explanations, including whether marijuana use may reinforce psychiatric symptoms or increase the risk of developing a psychiatric illness later in life [18-21]. A full understanding of marijuana use and its potential adverse effects, however, will require further research.

The trend toward the use of marijuana for therapeutic purposes among adults raises questions regarding how this may influence young people's use of marijuana for similar reasons. Recent studies suggest that adolescents are aware that marijuana is sometimes used to gain relief from physical and psychological pain [22-24]. Furthermore, there is evidence suggesting that adolescents may be using marijuana for reasons that are analogous to adults who use marijuana for therapeutic reasons. For example, young marijuana users with coping motives report more stressful life events (e.g., death of a family member or friend), personal injury and illness than socially motivated marijuana users and non-users [8]. There is also indirect evidence that adolescents with mental health conditions might be seeking relief through marijuana use. In a sample of 992 adolescents in drug treatment programs in four U.S. cities, more than half had at least one co-morbid mental disorder. In total, 72.5% of these youth were dependent on marijuana [25]. Among youth entering outpatient treatment programs for cannabis use disorders, 76% were reported to have concurrent mental health conditions [26]. Finally, in a sample of homeless young people in the UK who used a variety of drugs including marijuana, participants were found to be self-medicating to deal with the stress and problems they encountered including depression, loneliness, and physical problems such as aches and pains [13].

As part of a larger study to understand the culture of frequent marijuana use among young people, we were struck by the extent to which some participants spontaneously described using marijuana to gain relief from symptoms. In order to develop these emergent findings, we conducted a focused ethnography in which we examined the ways in which youth use marijuana to seek relief.

## Methods

This study was designed to understand and describe adolescents' experiences in using marijuana for therapeutic reasons, and explore how their constructions of these experiences are influenced by social norms. Compared to other types of ethnographic studies, focused ethnographies occur on a smaller scale and seek to examine a specific problem or phenomenon [27]. Typically, focused ethnographies are time-limited, involve a limited number of participants drawn from a specific population who have experience and understanding directly related to the area of inquiry, and are conducted through selected episodes of participant observation and/or interview [28]. In this focused ethnography, both in-depth interviews and participant observation were employed.

We drew data from a larger ethnographic study of frequent marijuana use among adolescents conducted in two rural and one urban location in British Columbia (BC), Canada. In the study communities, as is the case in much of BC, marijuana is readily accessible to youth despite the fact that it is illegal to grow, sell or possess. The use of marijuana for medical reasons is legally supported in Canada in limited circumstances; individuals meeting the criteria are provided with cannabis or given a license to grow a limited quantity for personal use.

Ethical approval for this study was obtained from the University Behavioral Research Ethics Board. Given the sensitivity of the topic and because we successfully argued that teens were able to provide consent for research participation, we did not require parental consent. As a courtesy, we provided the youth with a parent/guardian information letter which outlined the study's focus as pertaining to attitudes about marijuana use in general. Participants were told that they could take the letter home if they so chose. Prior to the interviews, written consent was obtained from the participants. Confidentiality was ensured at the outset and participants were informed that all identifying information would be removed from the data.

### Sample

In the larger study, participants were recruited by means of information fliers posted at high schools which invited youth to share their "views on marijuana use and teens." Youth who expressed interest in participating were screened for eligibility by the research team. Eligibility criteria included being 13–18 years of age and reporting having smoked marijuana at least once in the previous week. In total, 63 young people participated in the study. Although many youth described "feeling better" after they smoked marijuana, a subset [n = 20] explicitly described experiences of using marijuana on a regular basis specifically to manage, reduce or eliminate unpleasant and uncomfortable feelings or other health problems. They constructed marijuana as a treatment for health problems, often suggesting it had significantly greater benefit than other medical treatments they had been offered. None of these students were legally provided with cannabis for medical treatment or given a licence to grow marijuana for medicinal use. Characteristics of this sub-set of participants are presented in Table 1. The majority of youth using marijuana for relief were male, and the average age of initiation of marijuana was 13 years of age. Youth in this subset were of diverse ethnic backgrounds. Most [n = 12] indicated that they were "Canadian" or "Caucasian; " More specifically, 2 participants identified as First Nations, 6 individuals were part First Nations, 3 of UK descent and 3 were of European background including Italian, Croatian, and Ukrainian. Compared to those who used marijuana for the purpose of relief, those recruited to this study who smoked marijuana for recreational purposes (n = 43) smoked marijuana less frequently (average of 11 days in the last 30 days) and used marijuana more often with others.

**Table 1:** Description of participants who smoke marijuana for relief (n = 20)

*Gender*	
Female	7 (35%)
Male	13 (65%)
*Age (years)*	X = 16 yrs (range 14–18 yrs)
*Age of initiation (years)*	X = 13 yrs (range = 10–16 yrs)
*Frequency of use (days)*	X = 2 days/mo (range = 2–31 days/mo)
*Number of times/day*	1 – > 5 times/day
*Time of day of first use*	
Morning	4 (20%)
Afternoon	11 (55%)
Evening	5 (55%)
*Marijuana use when alone*	yes = 16 (80%)
Reasons for use*	
Depression	6
Stress/anxiety	12
Sleep problems	9
Focus/concentration	3
Physical pain	5

* some participants used marijuana for more than one reason

### Data Collection

The primary source of data was in-depth, semi-structured interviews with youth to glean accounts of their experiences with marijuana. We used a short questionnaire prior to beginning the qualitative interview to collect demographic data and to assess history of marijuana use and current use. The questionnaire included items related to marijuana use including age of initiation, use in the last month, frequency and quit attempts. We also collected data on the time of day that individuals usually used marijuana.

The interviews were conducted using an interview guide. Broad discussion categories included: history and pattern of use, the reasons for their use, what they knew about marijuana, the sources of that information as well as contextual factors related to their use. Open-ended questions were posed in relation to each of these topics, as required during the interviews. Many of these youth were at ease when talking about their use of marijuana and needed little prompting. When youth described the use of marijuana to help them feel better, participants were asked to elaborate further on their experiences.

The tape-recorded interviews took place in privacy within the school setting and lasted from 1 to 2 hours. Participants were offered a $20 honorarium. Field notes were used to record impressions of responses to the interview and the interviewer's assessment of the quality of the interview. In addition, field notes were maintained to record pertinent observations within the school and in the larger community (e.g., noting school policies regarding marijuana use at school and the presence of graffiti related to marijuana in close proximity of the school; visiting favourite outdoor settings where some indicated that they preferred to smoke marijuana along with hemp shops where they purchased pipes and bongs and other paraphernalia).

### Data Analysis

All data including transcribed interviews and field notes were reviewed several times by the research team paying close attention to young people's descriptions of experiences with the use of marijuana to address uncomfortable feelings or health problems, and the circumstances that surrounded this use. Close readings of the interviews by the investigative team involved highlighting potentially important comments, raising questions about the data, and identifying prominent dimensions of participants' experiences. During team meetings, interview data were discussed and emergent categories were identified to capture experiences related to marijuana use. These categories were organized into a coding framework and used to code the data. All coding was completed by one of the authors who completed a majority of the interviews (BMM). To code the data, we used [29] the NVivo software program designed for qualitative analysis of textual data. The program was also used to retrieve data coded under each category for a more nuanced analysis by the investigative team. Through reflective questioning of these data and detailed comparisons, themes were identified and discussed in team meetings.

## Results

### The Context of Using Marijuana for Relief

The teens situated their use of marijuana for relief of health problems in the context of difficult life events and illness experiences marked by a lack of supportive family networks, unexpected and sometimes traumatic losses of close friends or family members, and difficulties at school. Many indicated that they had few people to turn to help them; for some their parents were having difficulty coping with their own situations of unemployment, substance use, and marriage breakdowns and offered little support. Those living in households with a parent and step-parent had difficulty coping with unresolved feelings towards their estranged biological parent. Finally, several teens who made frequent moves with their families experienced social isolation at school and were subject to being bullied and teased.

Experiences with the medical system to address their health problems consistently fell short of the teens' expectations; their problems were either not taken seriously or the solutions offered were not helpful. For example, youth who reported they had been prescribed drugs such as Ritalin, Prozac or sleeping pills, stopped using them because they did not like how these drugs made them feel or found them ineffective. Despite visits to doctors, prescribed treatments and, for a few, hospitalizations, many of these teens perceived that they did not receive the help they needed from doctors.

A final contextual feature to these teens' lives were their observations of others' use of marijuana to deal with difficult circumstances or symptoms, including, in a few cases, parents and other significant adults in their life. For example, one young man reported that his mother was using marijuana while receiving cancer treatment. As he observed, "It helped her sleep and calmed her down." Others described how they were given advice from other teens about how marijuana could "help." Together these circumstances created a context where teens routinely turned to marijuana to manage physical and psychological problems in their lives. Marijuana was readily available, used by others in their network, and was perceived to provide an effective solution not offered to them from the medical system.

### Regular Relief:Patterns of Using Marijuana for Persistent Problems

Most of the participants who consistently used marijuana for relief, smoked it when alone, often several times a day. For some, their day began and ended with using marijuana; they smoked before leaving home for school and prior to going to bed. Some indicated that they needed to smoke marijuana during the school day to manage symptoms, and when this occurred it was often in the company of friends. A few participants smoked marijuana for relief in adult company that included relatives and "older" friends who supported their need to use marijuana to manage symptoms.

There were two patterns of marijuana use for relief: intermittent and chronic. With intermittent use, youth routinely relied on marijuana to deal with short-term problems such as stressful situations or limited periods of physical pain. One 14 year old male described non-daily use occurring whenever he had a "really bad day." In the case of chronic use, daily marijuana was used for the relief of identified conditions such as depression, ADHD and to routinely settle at night or manage sleeplessness. Young people's descriptions of marijuana use for relief were imbued with language common to using pharmaceuticals. A number of these youth indicated how they carefully titrated their intake; others described their use as "moderate," involving a "few puffs," or just a "certain amount." Through experience, they had learned to hone ways of using the right amount of marijuana to achieve a state of relief. As one male elaborated, he regulated his intake by mixing his marijuana with tobacco so as to get "just enough" marijuana to relieve regular states of agitation and high levels of stress. Along with skills at monitoring their intake of marijuana, these youth confidently shared in-depth knowledge of the strength and associated effects of different strains of marijuana.

### Explaining the Need to Use Marijuana for Relief

The young people in the sub-sample were particularly articulate in describing their "need" to use marijuana. They were adamant and confident that marijuana provided relief from their health problems. The decision to smoke marijuana was stated in a straight forward fashion (e.g., "I started it to make myself feel better") and justified because they had a "reason for it." Participants also framed their marijuana use in a positive manner; in so doing, gave credence to the claim that this was the right course of action. As one girl elaborated on her daily use, "Pot helps me be me." Several described unpleasant physical sensations such as feeling "jittery" associated with the absence of marijuana. For these youth, regular marijuana use allowed them relief from these unpleasant symptoms so that they were able to feel "normal." One 18-year old male who used marijuana everyday indicated, "If anything, it makes you more normal." Of note, he had first started to use marijuana at the age of 13, and smoked it regularly for 5 years typically 4 times a day.

For these youth, the purpose of smoking marijuana was not specifically about getting high or stoned, nor was marijuana used for "pleasure." In fact, participants tended to differentiate their own use from peers who were recreational users who smoked marijuana when they were "partying" or "socializing." As one 16-year old male described his use, "I don't get a strong sense of euphoria, I just calm down a bit, that's just how it is for me." However, there were a few instances when female participants did smoke "to get high" for the purpose of "escaping reality," a strategy used to remove themselves temporarily from the challenging circumstances that accompanied their daily lives. The participants also distinguished themselves from the "stoner" stereotype, whose preferred activities were watching movies or listening to heavy metal music while smoking marijuana.

Some explanations of using of marijuana to feel better were further bolstered with a focus on use for described "health" reasons. As one 16-year old female indicated, her daily use of marijuana was "more of a health thing, than to get high." She reflected on her history of "mild depression" and her difficulties with antidepressants that had resulted in insomnia and a loss of appetite. She suggested that these health issues would re-surface in the absence of marijuana, thereby providing solid rationale for her continued use of marijuana. One male situated his marijuana use within a perspective that medications are used to help deal with problems.

I bet you if I had never been put on Ritalin at a younger age, I might not have had the same opinion of drugs growing up, you know, because I was taught growing up that you take drugs to help you out with your problems, you know. [18 years, non-daily use]

Often, marijuana was compared to other substances in a way that suggested marijuana was the best option, further supporting ongoing use of marijuana for relief purposes. Some constructed marijuana as a "natural" substance that was preferable and considered "safer" than many pharmaceutical alternatives. One 14-year old female discovered that marijuana was a better option than dealing with the side effects of pharmaceuticals stating, "Well, my body, I have to be careful what pills I take. I have bad reactions to some medications. My body rejects it and I get really sick." Interestingly, one 18-year old who smoked twice a day on 21 days during the last month, went as far to describe himself as a "healthy marijuana user" adding, "It's not good for you, but then again, neither is MacDonald's and a lot of other things." The health claims in these descriptions served to explain the ongoing use of marijuana for relief.

### Painful Lives: Types of Symptoms and Distress Requiring Relief

In the interviews the teens directly linked their use of marijuana with the management of difficult feelings (including depression, anxiety and stress), sleep problems, problems with concentration and physical pain. Each of these will be described in the following sections.

#### Difficult Feelings

Although some teens described using marijuana to deal with instances of being angry, experiencing a significant disappointment (e.g., with exam results), being afraid, or to forget the past, the most frequent uses were associated with dealing with depression, and managing stress and anxiety.

##### Depression

Six participants indicated they were using marijuana specifically to deal with depression and several others reported knowing teens that were doing the same. Dealing with difficult personal circumstances was a common theme for this group of teens and was linked to the loss of significant people in their lives, a family history of depression, financial worries at home, "fights" with parents, abuse, and too much "shit" in their lives. Several reported receiving treatment for depression in the form of antidepressants and counseling, sometimes over extended periods, yet with little relief. For others, these options were not available in part because "nobody wanted to listen" to them. As a last resort, these teens had opted to try smoking marijuana. In a relatively short time, marijuana helped them to feel better about themselves, happier and more like the person they "wanted to be" as well as alleviate other problems associated with depression (poor appetite, difficulty concentrating, poor sleep).

Not all participants agreed about the use of marijuana for depression. One 16-year old male used marijuana to deal with his unhappiness surrounding the conflict between his mother and father, and worried that he might be using pot too frequently. He reasoned that being happy all of the time was not natural, and that there was nothing wrong with being sad and confused sometimes. As a result, he tried to limit using marijuana to weekends with friends. Others believed that marijuana should only be used for certain types of depression because of the possibility of becoming more depressed by smoking pot:

I think it depends on the level of depression that you have. If it's like depressed because you are sick, then pot is helping you. It's making you happier. But if you're depressed about killing yourself, I don't think that it's a good idea to smoke pot just because it could bring you down more. It's hard to say, though, it's different for every person, right? That just how it makes me feel. [Female, 17 years, daily use]

##### Stress and anxiety

The use of marijuana to manage stress and anxiety was described by 12 teens in our sample. Dealing with bullying at school, heavy demands of school work, taxing shifts at work, and just "giving as much as you can" along side difficult relationships with parents or guardians, and receiving threats from neighbors all took its toll on these youth. For some, these experiences contributed to high levels of stress and anxiety, and for others uncomfortable levels of anger – both were difficult to manage. Although some had friends they could turn to, marijuana provided an additional source of stress relief that was ready at hand.

Lots of people know me, know I do pot and they think that I'm a pot head but really the thing they don't realize is that I have a reason for it. It's for my stress and an antidepressant. I get really upset. It [pot] helps me feel better about myself, because you know people don't do that [help me], like my friend [name] can, but nobody else can. [Female, 14 years, non-daily use]

There was general agreement among the teens that marijuana calmed them down, and helped them feel "not so nervous" and "not so uptight about everything." One teen recognized, however, that despite the fact that marijuana could be a very effective stress reliever, it might not work for everyone:

Well as far as pot goes, the good thing is that it's definitely a stress reliever, hands down. I know lots of people who would be just a complete wreck if they weren't smoking pot but then there's also people who are a complete wreck because they do smoke pot, so it's kind of a hard thing. [Male, 16 years, non-daily use]

#### Sleep problems

Nine teens in our sample described using marijuana to help them sleep. The "trouble" they had with sleeping was a constant problem that many had experienced for years. One 16-year old, who also experienced mild depression, indicated that she "stopped sleeping for two years." Not only did the problem affect their school performance, but it was deeply disturbing to them. As another female described,

I have a really hard time sleeping. I can lay there for about four to five hours, just laying there. And I just finally had it, and I just feel like screaming I don't want to wake anyone up. So I go downstairs and ask my gran or my brother [for some marijuana] or I have a roach or two sitting around. [16 years, non-daily use]

Although one teen indicated that she had spoken to her mother about her problems sleeping, others indicated that the adults in their lives did not offer any support.

I have trouble going to sleep and waking up...My mum wanted to get the doctor to put me on sleeping pills but he said at such a young age it would cause like an addiction to them...I've had these problems since elementary school...I just, I can't go to sleep at night and then I like to sleep during the day. [Female, 14 years, non-daily use]

Many teens turned to pot and found almost immediate benefits in helping them sleep. Likened to a "magic sleeping pill" by one young male, the teens found it calmed their "busy minds," helped them relax and fall asleep quickly.

#### Focus/concentration

Three teens reported using marijuana to improve their concentration. They explained that they had difficulty focusing at school and that this affected their school performance. As one male explained:

Personally, I'm a very fast paced guy and my mind is always rushing, hard to gather my thoughts. I think a lot faster than I can speak. I get distracted very easily. In social studies last year, I would talk and wouldn't do any work. But if I had just a little bit of pot, I could really focus my work. I could sit there and I'd work all day and finish everything and have no homework and be done by the end of class. [16 years, non-daily use]

These young people believed they could "think better" when they used marijuana because it allowed them to focus their thinking, and, slow it down in a way that was preferable. All suggested that these cognitive changes were linked to improved school performance. One teen, who self-identified as a having "attention deficit, hyperactive disorder" shared the difficulties he experienced on Ritalin. He began smoking pot when he was 12 years of age and still on Ritalin.

Usually my mind is in over gear, right? I'm usually going about a mile a minute and my hands are moving way too fast, and I'm really fidgety. But if I have a puff of marijuana in a moderate use, by moderate I mean one to three to four puffs, depending on the quality....being toned down a bit I find really helps me....If I try to do homework at home without smoking pot, I just can't focus. I'll be looking at my schoolwork and for me with my ADHD this is how it's always been for me. Like school was just a constant story of this scenario before I smoked marijuana. [17 years, non-daily use]

#### Physical Pain

Five teens indicated they used marijuana to obtain pain relief, and several others shared similar stories about other youth. One male used marijuana to deal with pain associated with rehabilitation after a muscle injury, another used marijuana following an accident where he sustained 3^rd ^degree burns and yet another because of plates in his back due to a car injury. Others suggested that marijuana reduced muscle pain after a hard day of skiing and helped with headaches, and that girls used marijuana for menstrual cramps. One 17-year old male used it daily and explained that marijuana "numbs your systems or senses [and] relaxes your muscles."

### Considering the Risks of Using Marijuana for Relief

In spite of experiencing personal benefits from using marijuana for relief, some participants wrestled with their use of marijuana. One girl noted her own problematic use of marijuana that had quickly evolved into relying on it to deal with the regular stress in her life. As she pondered, she commented knowingly that it would be preferable to use it only when her stress level was "really" high.

I mean I started it, and I'm doing it for the wrong reasons...I think if I cut back and only did it when I was *really *stressed out or something, then, you know, really cut back, I think it would be okay. [14 years, non-daily use]

Although knowing that it was "harmful" to her body, she added that she found it difficult to quit using marijuana. Most youth were aware of the health consequences associated with marijuana use in general and their own use in particular. They noticed physical symptoms such as decreased stamina and shortness of breath with physical activities, while others worried about weakened immune systems and how it affected their energy level. Some recognized that they were addicted to marijuana. One male who had been using marijuana for six years framed it as something that he would address at a later date. "I'm trying to get through school and then worry about my dependency issue with marijuana."

Others noted that their marijuana use was linked to difficulties that they were having at school. One male concluded, "I think it brings marks down in one way and sometimes you don't understand things maybe as easily." Others recognized how their use had affected their memory. For a number of the participants, their knowledge of the risks of smoking marijuana was limited and, at times, incorrect. For example, as one 14-year old male who had started smoking marijuana in the past year to relieve muscle pain noted, "It's bad for your lungs, just it's 400 times lower than tobacco."

In what appeared to be an effort to minimize their use in the face of health risks, the teens emphasized that they were not using marijuana "in excess." One 18-year old summed up six years of using marijuana by saying, "I don't feel that I have a problem," adding that "it doesn't really have that many side effects." Some suggested that the benefits of smoking marijuana outweighed the risks. As noted, for those with difficulties sleeping at night, not being able to function the following day when sleep deprived was agonizing; marijuana use at night was preferable and provided a solution to that quandary. However, one male pondered both sides of his use of marijuana in dealing with his depression and was less optimistic:

Well, in some ways, it's helped me and some ways it hasn't. It's good when it's there, but when it's not, it kind of makes me sad. So it's hard like to try to keep up with staying happy all the time.[18 years, daily use]

Several participants made reference to the contradictions that they saw in their world regarding other licit substances and used that argument to make sense of and praise the benefits of marijuana over the risks.

And the thing is that if it's already used, they're already growing it for people that need it for medical help, then like why not.... Like no one has ever overdosed on marijuana, but people die everyday from alcohol, everyday from cigarettes and everyday from vast amounts of things that the government has legalized, but they just won't legalize marijuana for some reason. It's never killed anyone, never really hurt anyone, it saves people's lives and they could make a good amount of money from it and drop crime rates, why don't they do it? [Male, 14 years, non-daily use]

## Discussion

The findings of this study provide one of the first in-depth descriptions of youths' use of marijuana for non-recreational purposes, adding to the growing body of research on the use of drugs to self-medicate among young people. Teens involved in regular and long-term use of marijuana for relief constructed their use of marijuana as essential to feeling better or "normal" in situations where they perceived there were few other options available to them. Unlike the spontaneity typically involved in recreational use, these youth were thoughtful and prescriptive with their marijuana use – carefully monitoring and titrating their use to optimize its therapeutic effect. The findings also point to important contextual factors that further support youth's use of marijuana for relief that extend beyond the availability of marijuana and dominant discourses that construct marijuana as a natural product with medicinal properties.

Of key importance in the findings are the unmet health needs of these youth. Health issues such as depression, insomnia, and anxiety were significant problems that interfered with these youths' ability to function at school, maintain relationships with family and friends, and feel that they could live a normal life. The level of distress associated with these health concerns, along with the lack of effective interventions by heath care providers and family members appeared to leave them with few alternatives. Researchers have reported that when adolescents in rural communities experience barriers to seeking health care, they think they can take care of the problems themselves [30]. Similarly, our study participants believed that their best option was to assume responsibility for treating their problems by using marijuana. Unpleasant side effects with prescribed medications and long, ineffective therapies resulted in little hope that the medical system could be counted on as beneficial. In contrast, marijuana provided these youth with immediate relief for a variety of health concerns. Nevertheless, the regular use of marijuana put youth at risk. Cannabis use has been identified as a risk factor for mental illness such as psychosis, schizophrenia [21,31,32] and psychiatric symptoms such as panic attacks [33]. Teens who smoked marijuana at least once per month in the past year were found to be three times more likely to have suicidal thoughts than non-users [34], and there is evidence that exposure to cannabis may worsen depression in youth [35]. Marijuana use among youth has also been associated with other substance use and school failure [36]. What is interesting is that the findings of this study suggest that youth have little awareness of some of these risks; rather, some are using marijuana to counteract these very problems (e.g., depression, school failure). Teens' perceptions that their health concerns were not addressed suggest that more attention is needed to assess these issues and ensure that other options are available to them. Parents and health care providers need to make a concerted effort to not only understand the pressures and influences on youth [37], but also gain a better understanding of the effect of youths' health problems on their ability to engage in healthy lifestyle choices.

Underlying problems related to youth health concerns also need to be addressed. In many situations, the participants' symptoms appeared to be directly related to their life circumstances. Along with the challenges inherent in being an adolescent in today's complex world, some teens were also trying to deal with significant losses (death of a close friend or family member), extremely difficult family relationships, disappointments with friends, school and sports, and a fragile family and peer support network. The risk of substance use increases substantially when youth are attempting to deal with these kinds of situations in isolation. Although marijuana provided the youth with temporary relief, the underlying situation often went unattended – leading the teens into a regular pattern of use. Appropriate guidance and targeted support from counselors and health care providers must be sensitive to meeting the needs of youth as they work through such situations and life altering events. In addition, adults working with youth must find better ways to talk with young people about how they are coping with their health issues, including their marijuana use. Based on the experiences of youth in this study, there is a wide range of support that may benefit youth including counseling, stress management, social skills training, anger management, study skills, pain management, and sleep hygiene. The youth in this study had minimal access to these types of resources.

The influence of the policy environment in Canada related to medical marijuana cannot be dismissed. The youth in this study were familiar with medical marijuana and its sanctioned use among those with serious illnesses; some knew individuals in their social network who were medical marijuana users. In addition, we acknowledge that the availability of marijuana in the study settings provided teens with opportunities to try marijuana to relieve symptoms. In locales where it is more difficult to access marijuana and penalties for possession of marijuana are harsh, teens with similar symptoms may use other approaches.

Despite presenting themselves as being sophisticated users of marijuana, with a rich knowledge of marijuana acquired through direct experience, conversations and observations of others, the youth in our study did not appear to be well informed about the therapeutic use of marijuana. Targeted education for youth regarding the risks of marijuana and its appropriate use as a therapeutic agent is warranted, including the risks of legal sanctions. However, as Tupper [38] has suggested regarding drug education, fear-based approaches are unlikely to be effective when the reality of youths' observations and experiences suggest that few serious consequences stand in direct contrast to the "facts" teachers often provide. Alternative approaches are required that acknowledge the complexity of the issues that inform understandings of marijuana. Tupper suggests that drug education be framed using the metaphor of "drugs as tools" to allow "more nuanced understandings of the benefits and harms of drugs, depending on who is using them, in what circumstances, and for what purpose" (p. 235). This approach may be useful in education focused on marijuana.

This study was conducted in three locations in the province of British Columbia (BC) Canada and as such may not be generalized to other contexts. The province of BC is known for its illicit marijuana production [39]. And, in general the BC public is tolerant of marijuana use and support decriminalizing recreational use. In other contexts, teens might turn to other substances such as alcohol. The findings of this study provide a snapshot of these teens' use of marijuana. Further research is required to examine how this therapeutic use evolves over time.

## Conclusion

In summary, this study highlights youths' efforts to address their health problems and their experiences in using marijuana for relief. Marijuana may be perceived by some teens to be the only available alternative for those experiencing difficult physical or emotional problems when medical treatments have failed or when they lack access to appropriate health care. As has been noted in other studies of substance use [40], understanding why adolescents use particular substances is key in developing appropriate educational and intervention programs.

## Competing interests

The authors declare that they have no competing interests.

## Authors' contributions

JLB lead the data analysis, and conceptualized and participated in writing the manuscript. JLJ designed the larger study, participated in data analysis and the writing of the paper. BMM collected and analysed data, participated in writing of the manuscript. TM assisted with data management and contributed to the writing of the manuscript.

## References

[B1] Bonn-Miller MO, Zvolensky MJ, Bernstein A (2007). Marijuana use motives: Concurrent relations to frequency of past 30-day use and anxiety sensitivity among young adult marijuana smokers. Addict Behav.

[B2] Clark AJ, Ware MA, Yazer E, Murray TJ, Lynch ME (2004). Patterns of cannabis use among patients with multiple sclerosis. Neurology.

[B3] Coomber R, Oliver M, Morris C (2003). Using cannabis therapeutically in the UK: A qualitative analysis. J Drug Issues.

[B4] Ogborne AC, Smart RG, Adlaf EM (2000). Self-reported medical use of marijuana: A survey of the general population. CMAJ.

[B5] Ware MA, Doyle CR, Woods R, Lynch ME, Clark AJ (2003). Cannabis use for chronic non-cancer pain: Results of a prospective study. Pain.

[B6] Khantzian EJ (1985). The self-medication hypothesis of addictive disorders: Focus on heroin and cocaine dependence. Am J Psychiatry.

[B7] Simons J, Correia CJ, Carey KB, Borsari BE (1998). Validating a five-factor marijuana motives measure: Relations with use, problems, and alcohol motives. Journal of Counseling Psychology.

[B8] Brodbeck J, Matter M, Page J, Moggi F (2007). Motives for cannabis use as a moderator variable of distress among young adults. Addict Behav.

[B9] May L, Katzenstein D (2004). Healthy youth development: Highlights from the 2003 adolescent health survey.

[B10] Tjepkema M (2004). Use of cannabis and other illicit drugs. Health Rep.

[B11] Bolton J, Cox B, Clara I, Sareen J (2006). Use of alcohol and drugs to self-medicate anxiety disorders in a nationally representative sample. J Nerv Ment Dis.

[B12] Deykin EY, Levy JC, Wells V (1987). Adolescent depression, alcohol and drug abuse. Am J Public Health.

[B13] Klee H, Reid P (1998). Drug use among the young homeless: Coping through self-medication. Health.

[B14] Wilens TE, Adamson J, Sgambati S, Whitley J, Santry A, Monuteaux MC, Biederman J (2007). Do individuals with ADHD self-medicate with cigarettes and substances of abuse? Results from a controlled family study of ADHD. Am J Addict.

[B15] Glassner B (1987). Drugs in adolescent worlds: Burnouts to straights.

[B16] Beck AT, Wright FD, Newman CF, Liese B (1993). Cognitive therapy of substance abuse.

[B17] Chabrol H, Massot E, Mullet E (2004). Factor structure of cannabis related beliefs in adolescents. Addict Behav.

[B18] Amar MB, Potvin S (2007). Cannabis and psychosis: What is the Link?. J Psychoactive Drugs.

[B19] Cohen M, Solowij N, Carr V (2008). Cannabis, cannabinoids and schizophrenia: integration of the evidence. Aust N Z J Psychiatry.

[B20] Hall W, Degenhardt L (2007). Prevalence and correlates of cannabis use in developed and developing countries. Curr Opin Psychiatry.

[B21] Moore THM, Zammit S, Lingford-Hughes A, Barnes TRE, Jones PB, Burke M, Lewis G (2007). Cannabis use and risk of psychotic or affective mental health outcomes: A systematic review. Lancet.

[B22] Menghrajani P, Klaue K, Dubois-Arber F, Michaud P (2004). Swiss adolescents' and adults' perceptions of cannabis use: A qualitative study. Health Educ Res.

[B23] Plancherel B, Bolognini M, Stephan P, Laget J, Chinet L, Bernard M, Halfon O (2005). Adolescents' beliefs about marijuana use: A comparison of regular users, past users and never/occasional users. J Drug Educ.

[B24] Warner J, Room R, Adlaf EM (1999). Rules and limits in the use of marijuana among high-school students: The results of a qualitative study in Ontario. J Youth Stud.

[B25] Grella CE, Hser YI, Joshi V, Rounds-Bryant J (2001). Drug treatment outcomes for adolescents with comorbid mental and substance use disorders. J Nerv Ment Dis.

[B26] Tims FM, Dennis ML, Hamilton N, Buchan BJ, Diamond G, Funk R, Brantley LB (2002). Characteristics and problems of 600 adolescent cannabis abusers in outpatient treatment. Addiction.

[B27] Speziale HJS, Carpenter DR (2006). Qualitative research in nursing: Advancing the humanistic imperative.

[B28] Muecke M, Morse J (1994). On the evaluation of ethnographies. Critical issues in qualitative research methods.

[B29] QSR International Pty. Ltd (2006). NVivo Qualitative Data Analysis Software, Version 7.

[B30] Elliott BA, Larson JT (2004). Adolescents in mid-sized and rural communities: Foregone care, perceived barriers, and risk factors. J Adolesc Health.

[B31] Fergusson DM, Horwood LJ, Ridder EM (2005). Tests of causal linkages between cannabis use and psychotic symptoms. Addiction.

[B32] Patton GC, Coffey C, Carlin JB, Degenhardt L, Lynskey M, Hall W (2002). Cannabis use and mental health in young people: cohort study. BMJ.

[B33] Zvolensky MJ, Bernstein A, Sachs-Ericsson N, Schmidt NB, Buckner JD, Bonn-Miller MO (2006). Lifetime associations between cannabis, use, abuse, and dependence and panic attacks in a representative sample. J Psychiatr Res.

[B34] Greenblatt J (1998). Substance Abuse and Mental Health Services Administration. Adolescent self-reported behaviors and their association with marijuana use.

[B35] Office of National Drug Control Policy, Executive Office of the President (2008). Teen marijuana use worsens depression: An analysis of recent data shows "self-medicating" could actually make things worse.

[B36] Health Canada (2005). Health Canada's marihuana supply.

[B37] Substance Abuse and Mental Health Services Administration (2007). 2006 National Survey on Drug Use and Health.

[B38] Tupper KW (2008). Drugs, discourses and education: A critical discourse analysis of a high school drug education text. Discourse: Studies in the Cultural Politics of Education.

[B39] Mulgrew I (2005). Bud Inc: Inside Canada's marijuana industry.

[B40] Boys A, Marsden J, Strang J (2001). Understanding reasons for drug use amongst young people: a functional perspective. Health Educ Res.

